# Sports-Related Injuries in Adolescent Athletes: A Systematic Review

**DOI:** 10.7759/cureus.49392

**Published:** 2023-11-25

**Authors:** Mohammed A Al-Qahtani, Mansoor A Allajhar, Ahmed A Alzahrani, Mohammad A Asiri, Abdulaziz F Alsalem, Salha A Alshahrani, Naif M Alqahtani

**Affiliations:** 1 Family Medicine, Riyadh Third Health Cluster, Riyadh, SAU; 2 Epidemiology and Public Health, General Directorate of Health Affairs in Assir Region, Assir, SAU; 3 Epidemiology and Public Health, General Directorate of Health Affairs in Assir Region, Abha, SAU

**Keywords:** sports-related injuries in adolescents, adolescents, adolescent athletes, sports-related injuries, athletes

## Abstract

Sports participation among adolescents is increasing, offering numerous health benefits and exposing them to the risk of sports-related injuries. This paper aims to understand that the prevalence and risk factors associated with these injuries are crucial for effective injury prevention and the overall well-being of adolescent athletes.

This systematic review synthesizes the existing literature on sports-related injuries in adolescent athletes. A comprehensive search was conducted, yielding 11 relevant studies. The studies were analyzed to determine the prevalence of injuries and identify associated risk factors. A qualitative synthesis of the findings was performed.

The included studies collectively highlight the significant burden of sports-related injuries among adolescent athletes, with prevalence rates ranging from 34.1% to 65%. Specific risk factors associated with these injuries include body mass index (BMI), physical activity patterns, age, gender, sport type, previous injuries, and training practices. Obese adolescents, those engaged in excessive weekly practice hours, younger athletes, and females were found to be at higher risk. Certain sports, such as soccer and football, exhibited higher injury rates.

Sports-related injuries in adolescent athletes are a multifaceted issue influenced by various factors. Tailored injury prevention strategies are essential, considering the specific needs of adolescent athletes in different sports and age groups. Interventions should encompass physical and educational components, emphasizing proper warm-ups, protective equipment use, and injury prevention education. Longitudinal studies and standardized injury reporting systems are needed to monitor injury trends and evaluate prevention strategies effectively. This systematic review contributes to our understanding of sports-related injuries in adolescent athletes and underscores the importance of evidence-based injury prevention efforts.

## Introduction and background

Sports are crucial for adolescents' physical, mental, and social development, fostering discipline, teamwork, and a healthy lifestyle. However, sports-related injuries among adolescent athletes have become a pressing concern [1,2].

Adolescent athletes represent a unique demographic in sports medicine, characterized by rapid growth, changes in musculoskeletal development, and a desire for competitive achievement. The risk of sports-related injuries in this age group is particularly pronounced, leading to physical pain, discomfort, and potential disruption of academic and social functioning. The psychological impact of injury on young athletes, including stress, anxiety, and depression, is also a growing concern [3,4].

Healthcare professionals, coaches, parents, and governing bodies in sports have recognized the importance of addressing sports-related injuries in adolescent athletes. Multifactorial influences on sports-related injuries in adolescents include biomechanical factors, psychosocial factors, and gender disparities in injury rates and patterns [5]. Prevention strategies such as injury screening programs, strength and conditioning interventions, and educational initiatives have been proposed to mitigate the incidence of sports-related injuries in adolescents. However, the effectiveness and feasibility of these strategies remain topics of ongoing research and debate [6].

This systematic review aims to contribute to our understanding of the problem by synthesizing existing knowledge, highlighting gaps in the literature, and offering insights that can inform injury prevention and management strategies.

## Review

Methodology

Study Design

This systematic review follows the Preferred Reporting Items for Systematic Reviews and Meta-Analyses (PRISMA) guidelines to ensure transparency and rigor in the review process.

Search Strategy

A systematic and exhaustive search was conducted in electronic databases to identify relevant studies. The following databases were searched: PubMed/MEDLINE, Scopus, Web of Science, and Google Scholar. The search strategy included a combination of controlled vocabulary terms (e.g., Medical Subject Headings) and free-text keywords related to sports-related injuries, adolescents, and risk factors. The search strategy was adapted to each database's syntax and subject headings.

Inclusion Criteria were identified as studies published in peer-reviewed journals, studies conducted on adolescent athletes (aged 10-19 years), studies reporting the prevalence of sports-related injuries and/or associated risk factors, and studies with full-text availability in English. Exclusion Criteria included studies not focused on adolescent athletes, studies without available full text, studies published in languages other than English, and studies not reporting sports-related injuries or risk factors.

Study Selection

Two independent reviewers screened the search results based on the titles and abstracts to identify potentially relevant studies. The full texts of these potentially relevant studies were then reviewed to assess eligibility based on the inclusion and exclusion criteria. Any discrepancies between reviewers were resolved through discussion and, if necessary, consultation with a third reviewer.

Data Extraction

Two reviewers performed data extraction independently using a standardized data extraction form. The following information was extracted from each included study: Study details: author(s), publication year, study design, country, participant characteristics: age range, gender, sample size, injury characteristics: type of injury, anatomical location, injury mechanism, prevalence data: incidence rates, percentages, and relevant statistics, risk factors: variables associated with increased or decreased risk of injury, and methodological quality: assessment of study quality and risk of bias.

Data Synthesis

A qualitative synthesis of the included studies was conducted to analyze the prevalence of sports-related injuries and the associated risk factors. Findings were organized, summarized, and compared across studies to identify patterns and trends. Any discrepancies or contradictions in the literature were discussed, and efforts were made to explain the reasons behind these inconsistencies.

Ethical Considerations

Ethical approval was not required since this systematic review involved the analysis of published data and did not involve primary data collection from human subjects.

Results

The systematic review followed the PRISMA (Preferred Reporting Items for Systematic Reviews and Meta-Analyses) guidelines to ensure a rigorous and transparent search and selection process. The initial database search yielded a total of 1,623 potentially relevant articles. After removing duplicates, 854 articles remained for title and abstract screening. After screening, 98 full-text articles were assessed for eligibility based on the inclusion criteria.

**Figure 1:**
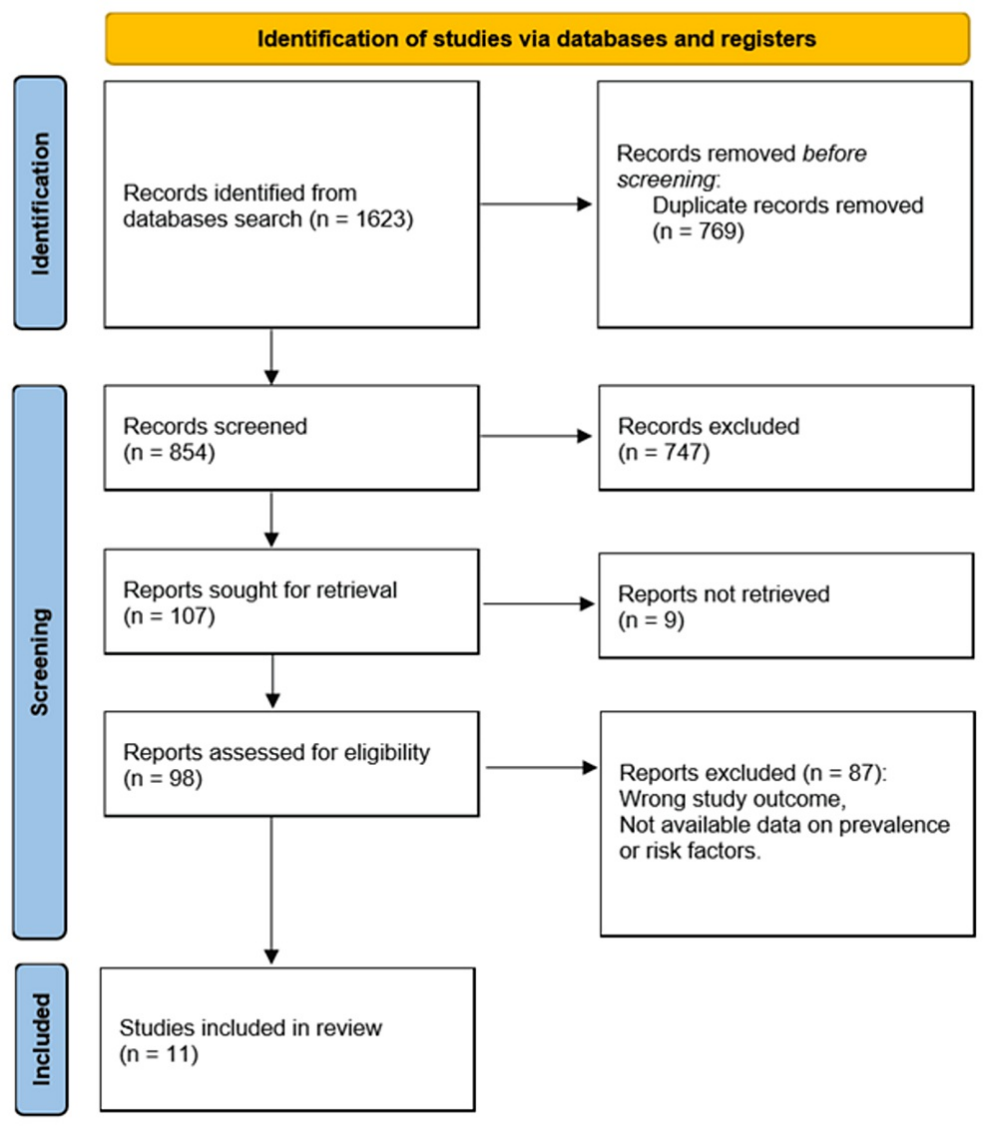
PRISMA Flow Diagram

Ultimately, 11 studies were included in the qualitative synthesis, as they met the predefined criteria for relevance and quality. The reasons for exclusion during the full-text review phase included insufficient data on sports-related injuries in adolescent athletes and studies that did not align with the primary research questions of this systematic review. The PRISMA flow diagram illustrating the selection process is presented in Figure 1.

Qualitative Data Synthesis

Table 1 illustrates the characters and findings of the eleven included studies. Prieto-González et al. conducted a prospective injury surveillance study involving 498 adolescent athletes aged 14 to 21. Their findings revealed that 40.4% of participants suffered injuries in 2019. The average injury rate was 2.64 per 1000 hours of participation, with soccer having the highest injury rate at 7.21. The most common injuries included lumbar muscle strains, ankle sprains, and bone fractures. Noteworthy risk factors associated with higher injury rates included greater weekly practice hours, the absence of warm-up routines, inadequate sports facilities, and younger age (14-17 years). These findings underscore the importance of addressing modifiable risk factors to reduce injury rates among adolescent athletes [5].

**Table 1:** Characters and findings of the included studies (n=11).

Study ID	Study Design	Population Information	Main Findings
Prieto-González et al., 2021 [5]	Prospective Injury Surveillance	498 adolescent athletes aged 14 to 21	40.4% of participants suffered an injury in 2019, average injury rate of 2.64 per 1000 hours. Soccer had the highest rate (7.21). Lumbar muscle strains, ankle sprains, and bone fractures were common. Factors associated with higher injury rates included greater practice hours, lack of warm-ups, inadequate facilities, ages 14-17, and more.
Richmond et al., 2013 [7]	Secondary Analysis of Surveys	4,339 students aged 12-19	Obese adolescents had a 34% increased risk of sports injury. Higher risk with increasing hours of play, Caucasian ethnicity, and higher sporting level.
Richmond et al., 2016 [8]	Prospective Study	1,040 adolescents aged 11-15	Overweight/obese adolescents had increased risk of time loss and knee injury. High waist circumference associated with time loss injury.
Habelt et al., 2011 [9]	Retrospective Analysis	4,468 adolescent injuries over 10 years	Football (31.13%), handball (8.89%), and school sports (8.77%) most common. Lower extremities (68.71%) most affected.
LeBrun et al., 2018 [10]	Analysis of Surveys	21,858 males and 24,691 females from 14 countries	Estimated over 23 million African adolescents sustained sports injuries annually.
Sreekaarini et al., 2014 [11]	Prospective Study	461 athletes aged 11-19	Prevalence of sports injuries was 65%. Male gender, age, psychological issues, previous injuries correlated with injury.
O'Connor et al., 2017 [12]	Descriptive Epidemiology	High school athletes during 2011-2014	Overall, 2004 sport-related concussions (SRCs) reported. Football had the highest SRC rate.
Leppänen et al., 2017 [13]	Prospective Study	387 young basketball and floorball players	Injury incidence 1.51 injuries/1,000 hours. Knee (35%) and lower back (21%) most affected.
Martínez-de-Quel-Pérez et al., 2019 [14]	Prospective Study	1,050 students aged 13.9 ± 1.3 years	0.30 injuries/student during teacher-supervised activities. Higher incidence at age 13 and in girls.
CDC, 2006 [6]	Data Analysis	High school athletes during 2005-06	An estimated 1.4 million injuries occurred at a rate of 2.4 injuries per 1,000 athlete exposures.
Sheu et al., 2016 [15]	Analysis of National Health Interview Survey	Persons aged 5 years and over during 2011-2014	Average annual estimate of 8.6 million sports- and recreation-related injury episodes. General exercise most frequent activity associated with injuries.

Richmond et al. examined the relationship between body mass index (BMI) and adolescent sports injuries. They found that obese adolescents had a 34% increased risk of sports injuries compared to those of healthy weight. Additionally, they observed a higher risk of injury with increasing hours of sports participation. The study highlighted the significance of BMI as a potential risk factor for sports-related injuries in adolescents [7].

Building on their previous work, Richmond et al. investigated the role of BMI and waist circumference (WC) as risk factors for sports injuries in adolescents. Their results indicated an increased risk of time loss and knee injuries among overweight/obese adolescents. High WC was also associated with a higher risk of time loss injuries. These findings emphasize the relevance of body composition measurements in assessing injury risk among adolescent athletes [8].

Habelt et al. conducted a retrospective analysis of 4,468 adolescent injuries over ten years. They identified that football (31.13%), handball (8.89%), and school sports (8.77%) were the most common activities associated with injuries. Lower extremities were involved in the majority (68.71%) of cases, with knee problems being particularly prevalent (29.79%). The study emphasized the importance of understanding injury patterns in different sports and the role of school-based activities in injury occurrence [9]. LeBrun et al. estimated the burden of sports injuries among African adolescents. Their findings indicated that over 23 million African adolescents sustained sports injuries annually. This study highlighted the substantial impact of sports injuries on the African adolescent population, emphasizing the need for injury prevention efforts in this region [10].

Sreekaarini et al. assessed the prevalence of sports injuries in adolescent athletes and identified associated risk factors. They reported a prevalence of sports injuries of 65% among the study participants. Male gender, age, psychological issues, previous injuries, and BMI were significantly correlated with the occurrence of injuries. These results underscored the multifactorial nature of adolescent sports injuries [11].

O'Connor et al. conducted a descriptive epidemiological study focusing on sport-related concussions (SRCs) among high school athletes. They reported 2,004 SRCs among 27 high school sports, with football having the highest SRC rate. The study revealed that the rate of SRCs was higher during competition than in practice. Notably, they found sex differences in reported SRCs, with different patterns of player-to-player contact. This study emphasized the importance of understanding the epidemiology of SRCs in high school sports [12].

Leppänen et al. conducted a prospective study investigating overuse injuries in youth team sports. They reported an injury incidence of 1.51 per 1,000 hours of exposure, with the knee and lower back being the most commonly affected body regions. Female athletes had a higher incidence rate, and previous injuries and playing at an adult level were identified as strong risk factors. This study highlighted overuse injuries' prevalence and risk factors in young team sport athletes [13]. Martínez-de-Quel-Pérez et al. examined the epidemiology of sports injuries during activities performed under teacher supervision among high school students. They reported an average of 0.30 injuries per student during such activities. In addition, they observed higher injury incidence among 13-year-olds and female students. These findings underscored the need for injury prevention strategies, especially in school-based physical activities [14].

The Centers for Disease Control and Prevention (CDC) analyzed data from high school athletes during the 2005-2006 academic year. They estimated that approximately 1.4 million injuries occurred among high school athletes, with an injury rate of 2.4 injuries per 1,000 athlete exposures. This study provided a comprehensive overview of sports injuries among high school athletes in the United States, emphasizing the substantial injury burden in this population [6].

Sheu et al. analyzed data from the National Health Interview Survey to estimate the annual number of sports- and recreation-related injury episodes among individuals aged 5 years and over in the United States. They reported an average annual estimate of 8.6 million such injury episodes. General exercise was the most frequent activity associated with injuries. This study shed light on the broader perspective of sports and recreation-related injuries, extending beyond the adolescent population [15].

In summary, the findings from the included studies collectively demonstrate the prevalence of sports-related injuries among adolescent athletes and highlight various risk factors contributing to these injuries. The prevalence rates vary across studies, reflecting the complex interplay of age, gender, BMI, sport type, and injury prevention practices. These findings underscore the importance of targeted injury prevention strategies that consider the specific characteristics of adolescent athletes and the sports they engage in. Future research should continue to explore these factors to develop effective interventions aimed at reducing sports-related injuries in this vulnerable population.

Discussion

The issue of sports-related injuries in adolescent athletes is a topic of great concern due to the potential short- and long-term consequences that can arise. The scientific background surrounding these injuries involves several key aspects, including the growing participation in youth sports, the unique characteristics of adolescence, and the multifactorial nature of injuries [16].

However, there are inherent risks that come with sports participation. These risks include acute injuries such as sprains, strains, fractures, and contusions, as well as more serious injuries such as concussions and spinal cord injuries. In addition, overuse injuries such as tendinitis and stress fractures can occur due to the repetitive nature of certain sports activities [4,16]. The increasing participation of adolescents in organized sports has led to a larger population being exposed to the inherent risks associated with sports participation. While engagement in sports has several benefits, including physical activity promotion, skill development, and competition, it also poses potential risks [2]. Adolescence is characterized by dynamic physical and psychosocial changes that can influence injury susceptibility. Adolescents often exhibit risk-taking behaviors and may not fully comprehend the importance of injury prevention practices. Therefore, it is essential to consider these factors when designing injury prevention strategies [16].

Studies have reported varying prevalence rates of sports-related injuries among adolescent athletes [5-15]. These disparities can be attributed to differences in study design, participant demographics, sporting activities assessed, and injury definitions. Nonetheless, the studies underscore the significant injury burden in this population. Certain sports exhibit higher injury rates than others. For example, soccer was identified as having the highest injury rate, which aligns with the popularity of the sport among adolescents and the frequent physical contact involved. Similarly, football was highlighted as having the highest sport-related concussion rate, emphasizing the need for targeted concussion prevention strategies in this sport [17].

The included studies identified various risk factors associated with sports-related injuries in adolescent athletes. Understanding these risk factors is vital for tailoring injury prevention interventions effectively. For instance, BMI was found to be a significant risk factor, with obese adolescents having an elevated risk of sports injuries. Physical activity patterns also played a role, with higher injury rates associated with greater weekly practice hours, emphasizing the need for balanced training schedules. Age and gender were also identified as significant risk factors, highlighting the need for age and gender-specific injury prevention strategies [5,6,11]. It is noteworthy that specific sports exhibited higher injury rates. For instance, Prieto-González et al. (2021) [5] identified soccer as having the highest injury rate, which aligns with the popularity of the sport among adolescents and the frequent physical contact involved. Similarly, O'Connor et al. (2017) [12] highlighted football as having the highest sport-related concussion (SRC) rate, emphasizing the need for targeted concussion prevention strategies in this sport.

Adolescents aged 13 and below exhibited a higher likelihood of injuries, while female athletes had increased injury rates in some studies. These findings highlight the need for age and gender-specific injury prevention strategies. Sreekaarini et al. (2014) [11] show that the recurrence of injuries emphasizes the importance of managing and rehabilitating previous injuries effectively to prevent future occurrences. The specific sport played can influence injury risk, as demonstrated by the higher injury rates in certain sports like soccer [5] and football [12]. Training factors such as warm-ups, appropriate equipment use, and supervision by qualified coaches emerged as important considerations [5].

Implications and future directions

The findings from this systematic review carry several implications for practice and research. First, injury prevention efforts must consider the diverse risk factors identified, tailoring strategies to address the specific needs of adolescent athletes in different sports and age groups. Second, interventions should encompass both physical and educational components. Promoting proper warm-ups, emphasizing the importance of protective equipment, and providing injury prevention education can play pivotal roles in reducing injury rates. Third, longitudinal studies are warranted to track injury patterns and the long-term consequences of sports-related injuries in adolescent athletes. Such research can inform the development of evidence-based interventions and guidelines. Lastly, there is a need for standardized injury reporting and surveillance systems to facilitate the collection of comprehensive injury data, enabling researchers and healthcare professionals to monitor trends and evaluate the effectiveness of prevention strategies.

## Conclusions

Sports-related injuries in adolescent athletes are a complex and multifaceted issue influenced by various factors. This systematic review provided valuable insights into the prevalence and risk factors associated with these injuries. While the included studies revealed varying prevalence rates, they collectively underscored the substantial burden of injuries in this population. The identified risk factors, including BMI, physical activity patterns, age, gender, and sport type, emphasize the need for tailored injury prevention strategies. Future research should focus on longitudinal studies and standardized injury reporting to enhance our understanding of sports-related injuries in adolescent athletes and guide evidence-based prevention efforts.
